# Molecular Evidence for an Old World Origin of Galapagos and Caribbean Band-Winged Grasshoppers (Acrididae: Oedipodinae: *Sphingonotus*)

**DOI:** 10.1371/journal.pone.0118208

**Published:** 2015-02-18

**Authors:** Martin Husemann, Jan Christian Habel, Suk Namkung, Axel Hochkirch, Daniel Otte, Patrick D. Danley

**Affiliations:** 1 Department of Ecology and Ecosystem Management, Terrestrial Ecology Research Group, Technische Universität München, Freising-Weihenstephan, Bavaria, Germany; 2 Biology Department, Baylor University, Waco, Texas, United States of America; 3 General Zoology, Institute of Biology, Martin-Luther University Halle-Wittenberg, Halle (Saale), Saxony-Anhalt, Germany; 4 Department of Biogeography, Trier University, Trier, Rhineland-Palatinate, Germany; 5 Department of Biodiversity, Earth & Environmental Science, Academy of Natural Sciences of Drexel University, Philadelphia, Pennsylvania, United States of America; Onderstepoort Veterinary Institute, SOUTH AFRICA

## Abstract

Patterns of colonization and diversification on islands provide valuable insights into evolutionary processes. Due to their unique geographic position and well known history, the Galapagos Islands are an important model system for evolutionary studies. Here we investigate the evolutionary history of a winged grasshopper genus to infer its origin and pattern of colonization in the Galapagos archipelago. The grasshopper genus *Sphingonotus* has radiated extensively in the Palaearctic and many species are endemic to islands. In the New World, the genus is largely replaced by the genus *Trimerotropis*. Oddly, in the Caribbean and on the Galapagos archipelago, two species of *Sphingonotus* are found, which has led to the suggestion that these might be the result of anthropogenic translocations from Europe. Here, we test this hypothesis using mitochondrial and nuclear DNA sequences from a broad sample of Sphingonotini and Trimerotropini species from the Old World and New World. The genetic data show two distinct genetic clusters representing the New World Trimerotropini and the Old World Sphingonotini. However, the *Sphingonotus* species from Galapagos and the Caribbean split basally within the Old World Sphingonotini lineage. The Galapagos and Caribbean species appear to be related to Old World taxa, but are not the result of recent anthropogenic translocations as revealed by divergence time estimates. Distinct genetic lineages occur on the four investigated Galapagos Islands, with deep splits among them compared to their relatives from the Palaearctic. A scenario of a past wider distribution of *Sphingonotus* in the New World with subsequent extinction on the mainland and replacement by *Trimerotropis* might explain the disjunct distribution.

## Introduction

Oceanic archipelagos are natural laboratories for studying evolutionary processes [1]. The Galapagos archipelago, in particular, has provided significant insight into our current understanding of speciation [2–4]. Its remote location far off the coast of Ecuador and its well-known geologic history [5] provide a unique opportunity to study colonization and subsequent radiation processes. The islands in their current state developed less than 5 million years ago [5, 6, 7]. The ages of the central and western islands, however, are much younger and range between 0.5 and 2.5 my [8]. This variation in island age might influence patterns of divergence within the archipelago.

The location of the archipelago has determined the general colonization source: phylogeographic studies have shown that most animal and plant species endemic to the Galapagos Islands originated in South America and radiated after one or multiple colonization events [9–13]. Subsequent ‘island hopping’ led to further differentiation among island lineages [7, 13]. Hence, most organisms found in the Galapagos archipelago belong to Neotropic groups and very few studies have shown a direct relationship of Galapagos endemics to any Old World taxon (e.g. the Hemipteran *Nezara viridula* [14, 15]). However, for many Galapagos endemics no closely related taxa occur in the Old World. In the rare instance that Galapagos species have both New and Old World relatives, phylogeographic studies often neglect the Old World as possible colonization source.

Representatives of the grasshopper genus *Sphingonotus* Fieber, 1852 provide a rare case of Galapagos endemics for which representatives can be found in both the New and the Old World [16, 17]. This genus is among the most species-rich grasshopper genera worldwide [18, 19]. Its main centres of species richness and endemism are the Mediterranean, central and eastern Asia, but a limited number of species have been described from Australia, South Africa, the Caribbean and Galapagos [17, 20–22]. The genus contains many endemics with very limited geographic distributions [18], often endemic to islands [20, 22]. In North and South America the genus is replaced by the ecologically similar genus *Trimerotropis* Stål, 1873 [19, 23]. Both genera were until recently grouped in the same tribe (Sphingonotini) [23]. However, it has been demonstrated that they split some 35 million years ago [23]. The presence of *Sphingonotus* species in the Caribbean (*Sphingontous haitensis* (Saussure, 1861)) and Galapagos (*Sphingonotus fuscoirroratus* (Stål, 1861)) is puzzling as these archipelagos are far off the main distribution [24]. Only two other *Sphingonotus* species have been recorded from the New World, *Sphingonotus brasilianus* Saussure, 1888 and *Sphingonotus punensis* Dirsh, 1969. The types of *S*. *brasilianus* are lost (NHMW pers. com.) [25] and the description of the species is insufficient to judge the status of the species. Hence, we consider it as nomen dubium. *Sphingonotus punensis* from Puna Island close to the Ecuadorian coast is morphologically very similar to *S*. *fuscoirroratus* [26, 27] and thought to belong to the same species group. However, only a single female of the species is known [26]. *Sphingonotus fuscoirroratus* itself has a complex history. Originally two species (*S*. *trinesiotis* Snodgrass, 1902, *S*. *tetranesiotis* Snodgrass, 1902) with several subspecies were described from the Galapagos Islands [28], which later were synonymised [29]. This synonymy was subsequently confirmed by morphological analyses, including inner genitalia, as the island populations could not be separated [26]. Similarly, *S*. *haitensis* was originally split in three species (*S*. *haitensis*, *S*. *jamaicensis* Saussure, 1884, *S*. *cubensis* Saussure, 1884). However, currently, only a single species with two subspecies is considered valid [16]. Interestingly, both taxa have been connected to the European species *Sphingonotus caerulans* in the past due to extremely similar phallic structures [26] and on the basis of the outer morphology [16].

To study the reasons for this disjunct distribution pattern across both continents, we test three hypotheses using a wide geographic sampling and DNA sequences of two mitochondrial genes and a nuclear gene fragment. (i) The taxonomic assignment of the Caribbean and Galapagos species might be wrong and these species may be related to the New World genus *Trimerotropis*. (ii) It has been suggested that the occurrence of *Sphingonotus* in the Caribbean is the result of recent anthropogenic translocation of a European species [16]. (iii) Alternatively, their presence may be the result of ancient long-distance colonization from the Old World and may be the relict of a formerly wider distribution.

## Results

We sequenced a 651 bp long fragment of the Cytochrome Oxidase I (COI) gene for a total of 104 specimens. The alignment for the NADH Dehydrogenase subunit 5 (ND5) fragment consisted of 955 bp and 104 sequences. For the nuclear Histone 3 (H3) gene fragment 293 bp were sequenced for the same set of taxa (Table 1). The ND5 alignment had 401 variable sites (42.0%), 317 of which were parsimony informative. The COI alignment had 225 variable sites (34.6%), 200 of which were parsimony informative. H3 had 26 variable sites (8.9%), 18 of which were parsimony informative.

**Table 1:** Overview of all samples used for molecular analyses; given are sampling location, GPS-coordinates, date of sampling and respective Genbank accession numbers.

ID	Tribe	Genus	Species	Country	County/Island/City	Collector	Genbank accessions		
							COI	ND5	H3
T76	Chortophagini	*Chortophaga*	*viridifasciata*	USA, Texas	McLennan Co.	MH	JQ513034	JQ513132	JQ513175
T112	Cibolacrini	*Cibolacris*	*parviceps*	USA, Texas	Brewster Co.	MH	JQ513033	JQ513133	JQ513176
T25	Trimerotropini	*Circotettix*	*maculatus*	USA, California	Mono Co.	D. Ferguson	JQ513041	JQ513134	JQ513177
T26	Trimerotropini	*Circotettix*	*maculatus*	USA, California	Mono Co.	D. Ferguson	JQ513045	JQ513135	JQ513178
T10	Trimerotropini	*Circotettix*	*rabula*	USA, New Mexico	Sandoval Co.	D. Ferguson	JQ286519	JQ286651	JQ286578
T108	Trimerotropini	*Circotettix*	*rabula*	USA, Montana	Yellowstone Co.	R.D. Scott	JQ513044	JQ513136	JQ513179
T9	Trimerotropini	*Circotettix*	*rabula*	USA, New Mexico	Sandoval Co.	D. Ferguson	JQ286518	JQ286650	JQ286577
T150	Trimerotropini	*Circotettix*	*stenometopus*	USA, California	Glenn Co.	D. Ferguson	JQ513039	JQ513137	JQ513180
T23	Trimerotropini	*Circotettix*	*undulatus*	USA, California	Mono Co.	D. Ferguson	JQ513043	JQ513138	JQ513181
T24	Trimerotropini	*Circotettix*	*undulatus*	USA, California	Mono Co.	D. Ferguson	JQ513042	JQ513139	JQ513182
T15	Trimerotropini	*Conozoa*	*texana*	USA, New Mexico	Valencia Co.	D. Ferguson	JQ286500	JQ286632	JQ286567
K379	Sphingonotini	*Leptopternis*	*maculatus*	Tunisia	Ouesslatia	AH	JQ513074	JQ513140	JQ513183
K473	Sphingonotini	*Sphingoderus*	*carinatus*	Tunisia	Bou Hedma	AH	KJ923334	KJ923393	KP201145
K315	Sphingonotini	*Sphingonotus*	*caerulans*	France	Vergières / Crau	AH	JQ513068	JQ513142	JQ513185
K608	Sphingonotini	*Sphingonotus*	*caerulans*	Finland	Hanko Taktom	AH	JQ513067	JQ513143	JQ513186
K613	Sphingonotini	*Sphingonotus*	*caerulans*	Italy	Affi	S. Lötters	KJ923335	KJ923394	KP201146
K512	Sphingonotini	*Sphingonotus*	*canariensis*	Cape Verde	Maio	M. Lecoq	JQ513077	JQ513144	JQ513187
K403	Sphingonotini	*Sphingonotus*	*candidus*	Italy	Sardinia	Y. Görzig	JQ513066	JQ513145	JQ513188
K262	Sphingonotini	*Sphingonotus*	*corsicus*	France	Corse	F. Pahlmann	KJ923336	EU266719	KP201147
K90	Sphingonotini	*Sphingonotus*	*femoralis*	Niger	Tabourax	T. McNary	JQ513065	JQ513146	JQ513189
K383	Sphingonotini	*Sphingonotus*	*finotianus*	Tunisia	Enfida	AH	JQ513073	JQ513147	JQ513190
K456	Sphingonotini	*Sphingonotus*	*fuerteventurae*	Spain	Canary Islands, Fuerteventurae	AH, MH	JQ513071	JQ513148	JQ513191
K424	Sphingonotini	*Sphingonotus*	*fuscoirroratus*	Ecuador	Galapagos Islands, Floreana	D. Otte	KJ923337	KJ923395	KJ923386
K631	Sphingonotini	*Sphingonotus*	*fuscoirroratus*	Ecuador	Galapagos Islands, San Cristobal	D. Otte	KJ923338	KJ923396	KP201148
K632	Sphingonotini	*Sphingonotus*	*fuscoirroratus*	Ecuador	Galapagos Islands, Santa Cruz	D. Otte	KJ923339	KP201198	KP201149
T166	Sphingonotini	*Sphingonotus*	*fuscoirroratus*	Ecuador	Galapagos Islands, San Cristobal	D. Otte	KJ923340	KP201199	KP201150
T167	Sphingonotini	*Sphingonotus*	*fuscoirroratus*	Ecuador	Galapagos Islands, San Cristobal	D. Otte	KJ923341	KP201200	KP201151
T169	Sphingonotini	*Sphingonotus*	*fuscoirroratus*	Ecuador	Galapagos Islands, Floreana	D. Otte	KJ923343	KJ923397	KP201152
T170	Sphingonotini	*Sphingonotus*	*fuscoirroratus*	Ecuador	Galapagos Islands, Santa Cruz	D. Otte	KJ923344	KJ923398	KP201153
T171	Sphingonotini	*Sphingonotus*	*fuscoirroratus*	Ecuador	Galapagos Islands, Santa Cruz	D. Otte	KJ923345	KJ923399	KJ923387
T172	Sphingonotini	*Sphingonotus*	*fuscoirroratus*	Ecuador	Galapagos Islands, Santa Fe	D. Otte	KJ923346	KJ923400	KJ923388
T54	Sphingonotini	*Sphingonotus*	*fuscoirroratus*	Ecuador	Galapagos Islands, Santa Fe	D. Otte	KJ923349	KJ923401	KP201154
T56	Sphingonotini	*Sphingonotus*	*fuscoirroratus*	Ecuador	Galapagos Islands, Floreana	D. Otte	KJ923350	KJ923403	KP201155
T66	Sphingonotini	*Sphingonotus*	*fuscoirroratus*	Ecuador	Galapagos Islands, Floreana	D. Otte	KJ923351	KJ923404	KP201156
K14	Sphingonotini	*Sphingonotus*	*guanchus*	Spain	Canary Islands, Gran Canary	AH	JQ513064	EU266743	JQ513192
K638	Sphingonotini	*Sphingonotus*	*guanchus*	Spain	Canary Islands, Gran Canary	R. Bland	JQ513063	JQ513149	JQ513193
T178	Sphingonotini	*Sphingonotus*	*haitensis*	Dominican Republic	Prov. Independencia	A. Hilario	KP201141	KJ923405	KP201157
T179	Sphingonotini	*Sphingonotus*	*haitensis*	Dominican Republic	Prov. San Christobal	A. Hilario	KJ923354	KJ923406	KJ923390
T180	Sphingonotini	*Sphingonotus*	*haitensis*	Dominican Republic	Prov. San Christobal	A. Hilario	KJ923355	KJ923407	KJ923391
T184	Sphingonotini	*Sphingonotus*	*haitensis*	Dominican Republic	Prov. San Juan	H. Takizawa	KP201142	KJ923408	KP201158
T39	Sphingonotini	*Sphingonotus*	*haitensis*	Dominican Republic	Prov. Peravia	D. Perez, B. Hierro	KJ923356	KJ923409	KP201159
T40	Sphingonotini	*Sphingonotus*	*haitensis*	Dominican Republic	Prov. Pedernales	D. Perez, B. Hierro, R. Bastardo	KJ923357	KJ923410	KP201160
T41	Sphingonotini	*Sphingonotus*	*haitensis*	Dominican Republic	Prov. Pedernales	D. Perez, B. Hierro, R. Bastardo	KJ923358	KJ923411	KP201161
K651	Sphingonotini	*Sphingonotus*	*maroccanus*	Morocco	Ameskrout	MH	JQ513075	JQ513150	JQ513194
K616	Sphingonotini	*Sphingonotus*	*ningsianus*	China	unknown	unknown	JQ513060	JQ513151	JQ513195
K470	Sphingonotini	*Sphingonotus*	*octofasciatus*	Tunisia	Gafsa	AH	JQ513058	JQ513152	JQ513196
K351	Sphingonotini	*Sphingonotus*	*rubescens*	Spain	Canary Islands, Fuerteventurae	AH, MH	JQ513069	JQ513153	JQ513197
K510	Sphingonotini	*Sphingonotus*	*rubescens*	Cape Verde	Fopo	M. Lecoq	JQ513070	JQ513154	JQ513198
K5	Sphingonotini	*Sphingonotus*	*rugosus*	Spain	Canary Islands, Lanzarote	AH	KJ923359	EU266739	KP201162
K150	Sphingonotini	*Sphingonotus*	*savignyi*	Spain	Canary Islands, Gran Canary	AH	JQ513076	JQ513155	JQ513199
K214	Sphingonotini	*Sphingonotus*	*scabriculus*	Namibia	Otjiu	W. Schuett	JQ513061	JQ513156	JQ513200
K615	Sphingonotini	*Sphingonotus*	*tsinlingensis*	China	unknown	unknown	JQ513059	JQ513157	JQ513201
K227	Sphingonotini	*Thalpomena*	*caerulescens*	Morocco	Irhil-n’-Isemsiden	AH	JQ513057	JQ513158	JQ513203
K641	Sphingonotini	*Thalpomena*	*viridipennis*	Morocco	Imouzzer	MH, JCH	JQ513056	JQ513159	JQ513204
T27	Trimerotropini	*Trimerotropis*	*californica*	USA, New Mexico	Socorro Co.	D. Ferguson	KJ923360	KJ923412	KP201163
T28	Trimerotropini	*Trimerotropis*	*californica*	USA, New Mexico	Socorro Co.	D. Ferguson	JQ513048	JQ513160	JQ513205
T21	Trimerotropini	*Trimerotropis*	*cincta*	USA, New Mexico	Sandoval Co.	D. Ferguson	KJ923361	KJ923413	KP201164
T22	Trimerotropini	*Trimerotropis*	*cincta*	USA, New Mexico	Sandoval Co.	D. Ferguson	KJ923362	KJ923414	KP201165
T17	Trimerotropini	*Trimerotropis*	*cyaneipennis*	USA, New Mexico	Valencia Co.	D. Ferguson	JQ513040	JQ513161	JQ513206
T18	Trimerotropini	*Trimerotropis*	*cyaneipennis*	USA, New Mexico	Valencia Co.	D. Ferguson	KJ923363	KJ923415	KP201166
T3	Trimerotropini	*Trimerotropis*	*cyaneipennis*	USA, Arizona	Mojave Co.	D. Ferguson	KJ923364	KJ923416	KP201167
T4	Trimerotropini	*Trimerotropis*	*cyaneipennis*	USA, Arizona	Mojave Co.	D. Ferguson	KJ923365	KJ923417	KP201168
T104	Trimerotropini	*Trimerotropis*	*pallidipennis*	USA, Montana	Big Horn Co.	R.D. Scott	JQ286536	JQ286668	KP201169
T105	Trimerotropini	*Trimerotropis*	*pallidipennis*	USA, Montana	Big Horn Co.	R.D. Scott	JQ286539	JQ286671	JQ286598
T109	Trimerotropini	*Trimerotropis*	*latifasciata*	USA, Montana	Blaine Co.	R.D. Scott	KJ923366	KJ923418	KP201170
T110	Trimerotropini	*Trimerotropis*	*latifasciata*	USA, Montana	Blaine Co.	R.D. Scott	JQ513047	JQ513163	JQ513208
T111	Trimerotropini	*Trimerotropis*	*latifasciata*	USA, Montana	Blaine Co.	R.D. Scott	KJ923367	KJ923419	KP201171
T1	Trimerotropini	*Trimerotropis*	*maritima*	USA, Texas	McLennan Co.	MH, PDD	JQ286498	JQ286630	JQ286565
T2	Trimerotropini	*Trimerotropis*	*maritima*	USA, Texas	McLennan Co.	MH, PDD	KJ923368	KJ923420	KP201172
T52	Trimerotropini	*Trimerotropis*	*maritima*	USA, Texas	Bosque Co.	MH, PDD	JQ286497	JQ286629	JQ286564
T86	Trimerotropini	*Trimerotropis*	*maritima*	USA, Texas	Brewster Co.	MH	KJ923369	KJ923421	KP201173
T29	Trimerotropini	*Trimerotropis*	*melanoptera*	USA, New Mexico	Valencia Co.	D. Ferguson	KJ923370	KJ923422	KP201174
T30	Trimerotropini	*Trimerotropis*	*melanoptera*	USA, New Mexico	Valencia Co.	D. Ferguson	KJ923371	KJ923423	KP201175
T14	Trimerotropini	*Trimerotropis*	*modesta*	USA, Arizona	Coconino Co.	D. Ferguson	KJ923372	KJ923425	KP201176
T57	Trimerotropini	*Trimerotropis*	*modesta*	USA, Arizona	Cochise Co.	D.R. Swanson	KJ923373	KP201201	KP201177
T58	Trimerotropini	*Trimerotropis*	*modesta*	USA, Arizona	Cochise Co.	D.R. Swanson	KJ923374	KP201202	KP201178
T152	Trimerotropini	*Trimerotropis*	*occidentalis*	USA, California	Glenn Co.	D. Ferguson	KJ923375	KJ923426	KP201179
T153	Trimerotropini	*Trimerotropis*	*occidentalis*	USA, California	Glenn Co.	D. Ferguson	KJ923376	KP201203	KP201180
T116	Trimerotropini	*Trimerotropis*	*ochraceipennis*	Chile	Coquimbe	J. Pizarro	JQ286549	JQ286681	JQ286607
T117	Trimerotropini	*Trimerotropis*	*ochraceipennis*	Chile	Coquimbe	J. Pizarro	JQ286547	JQ286679	KP201181
T118	Trimerotropini	*Trimerotropis*	*ochraceipennis*	Chile	Coquimbe	J. Pizarro	JQ286546	JQ286678	KP201182
T119	Trimerotropini	*Trimerotropis*	*ochraceipennis*	Chile	Coquimbe	J. Pizarro	JQ286548	JQ286680	JQ286606
T128	Trimerotropini	*Trimerotropis*	*ochraceipennis*	Chile	Coquimbe	J. Pizarro	KJ923377	JQ286688	JQ286622
T130	Trimerotropini	*Trimerotropis*	*pallidipennis*	USA, Texas	Brewster Co.	MH	KP201143	JQ286690	KP201183
T140	Trimerotropini	*Trimerotropis*	*pallidipennis*	Mexico	El Coptal	D. Salas	JQ286533	JQ286665	KP201184
T141	Trimerotropini	*Trimerotropis*	*pallidipennis*	Mexico	Marquez	D. Salas	JQ286527	JQ286659	KP201185
T144	Trimerotropini	*Trimerotropis*	*pallidipennis*	Mexico	El Coptal	D. Salas	JQ286562	KP201204	KP201186
T156	Trimerotropini	*Trimerotropis*	*pallidipennis*	Mexico	Salamanca	D. Salas	JQ286522	JQ286654	JQ286581
T162	Trimerotropini	*Trimerotropis*	*pallidipennis*	Mexico	Salamanca	D. Salas	JQ286537	JQ286669	JQ286596
T163	Trimerotropini	*Trimerotropis*	*pallidipennis*	Mexico	Salamanca	D. Salas	JQ286535	JQ286667	JQ286594
T124	Trimerotropini	*Trimerotropis*	*pistrinaria*	USA, Texas	Whitney Co.	MH	KJ923379	KJ923427	KP201187
T31	Trimerotropini	*Trimerotropis*	*pistrinaria*	USA, New Mexico	Valencia Co.	D. Ferguson	JQ513046	JQ513165	JQ513210
T19	Trimerotropini	*Trimerotropis*	*pseudofasciata*	USA, Utah	Tooele Co.	D. Ferguson	KJ923381	KJ923428	KP201188
T20	Trimerotropini	*Trimerotropis*	*pseudofasciata*	USA, Utah	Tooele Co.	D. Ferguson	KJ923382	KJ923429	KP201189
T132	Trimerotropini	*Trimerotropis*	*saxatilis*	USA, Texas	Hill Co.	M. Hanitzsch	JQ286503	JQ286635	JQ286570
T133	Trimerotropini	*Trimerotropis*	*saxatilis*	USA, Texas	Hill Co.	M. Hanitzsch	JQ286502	JQ286634	KP201190
T154	Trimerotropini	*Trimerotropis*	*saxatilis*	USA, Missouri	unknown	A. Templeton	KJ923383	KJ923430	KP201191
T155	Trimerotropini	*Trimerotropis*	*saxatilis*	USA, Missouri	unknown	A. Templeton	KJ923384	KJ923431	KP201192
T68	Trimerotropini	*Trimerotropis*	*sp*	Argentina	Mendoza Prov.	V. Confalonieri	JQ286552	JQ286684	KP201193
T69	Trimerotropini	*Trimerotropis*	*sp*	Argentina	Mendoza Prov.	V. Confalonieri	JQ286555	JQ286687	KP201194
T70	Trimerotropini	*Trimerotropis*	*sp*	Argentina	San Luis Prov.	V. Confalonieri	JQ286554	JQ286686	KP201195
T71	Trimerotropini	*Trimerotropis*	*sp*	Argentina	San Luis Prov.	V. Confalonieri	JQ286553	JQ286685	JQ286611
T11	Trimerotropini	*Trimerotropis*	*verruculata suffusa*	USA, New Mexico	Sandoval Co.	D. Ferguson	KP201144	KJ923432	KP201196
T12	Trimerotropini	*Trimerotropis*	*verruculata suffusa*	USA, New Mexico	Sandoval Co.	D. Ferguson	KJ923385	KP201205	KP201197

**Fig 1:**
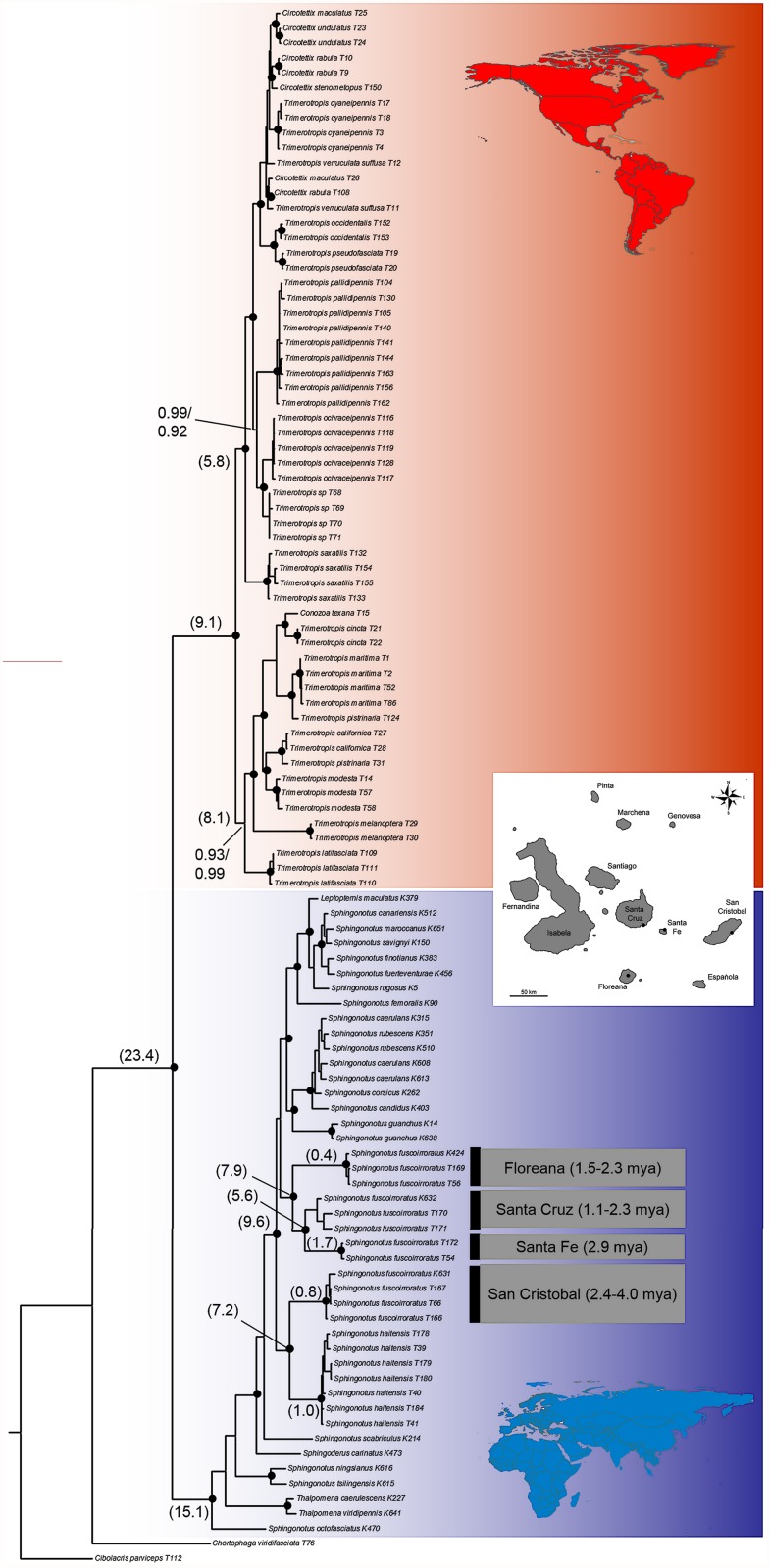
Phylogenetic tree resulting from Bayesian analysis of the combined data set of three genes. Red color indicates the New World Trimerotropini, blue are the Old World Sphingonotini. Black circles represent posterior probabilities ≥ 0.95 in both analyses. Numbers are posterior probabilities below 0.95 for at least one of the analyses (upper value from BEAST analysis, lower value from MrBayes analysis). The numbers in parentheses represent the divergence time estimates derived from the BEAST analysis. Only the values for main branches of interest are shown and no intraspecific values are presented. Estimates of minimum and maximum emergence times of the studied islands in parentheses next to island names were taken from Geist and colleagues [5].

We used two different phylogenetic reconstruction methods, MrBayes and BEAST, which both yielded similar groupings: a major split with high posterior probabilities (pp = 1 for both methods) was identified separating the New World Trimerotropini and the Old World Sphingonotini (Fig. 1). Within the Trimerotropini two groups were detected with high confidence (pp = 1 for both methods) corresponding to the chromosomal groups defined by White [30–32] and previously confirmed by Husemann and colleagues [23]. Further, within the Trimerotropini most species for which multiple individuals were sequenced were monophyletic, besides *Trimerotropis pistrinaria* Saussure, 1884 and some species of the genus *Circotettix* Scudder, 1876. *Sphingonotus haitensis* from the Dominicanian Republic and *S*. *fuscoirroratus* from four Galapagos Islands grouped within the Sphingonotini. Within the Sphingonotini *S*. *octofasciatus* (Serville, 1838), the genus *Thalpomena* Saussure, 1884 and the *Sphingonotus* species from China split basally from the other species in the group. The next split separates *Sphingoderus carinatus* (Saussure, 1888) from a group consisting of all other *Sphingonotus* species including *S*. *haitensis* and *S*. *fuscoirroratus*. The first taxon splitting off in this group is *S*. *scabriculus* Stål, 1876 from South Africa followed by the New World *Sphingonotus* species; *Sphingonotus fuscoirroratus* from San Cristobal groups together with *S*. *haitensis* in both analyses with high support (pp ≥ 0.99). The *S*. *fuscoirroratus* lineages from the other three islands form a second monophyletic group with the lineages from Santa Fe and Santa Cruz being sister clades. However, *S*. *fuscoirroratus* is not monophyletic in either analysis. The remaining *Sphingonotus* species from Eurasia and Africa branch off subsequently.

Both RASP analyses (S-DIVA and Bayes-Lagrange) yielded similar results suggesting an African origin for the Sphingonotini as a whole and a wider distribution (Africa and Galapagos) for the ancestral taxa of the New World *Sphingonotus* species (S1 Fig.). The molecular clock analyses dated the divergence between the two major clades (Trimerotropini from the New World and Sphingonotini from the Old World) at approximately 23.4 million years ago. The onset of the Trimerotropini radiation was dated at 9.1 million years ago. The Sphingonotini radiation was dated to be older with an age of 15.1 my. The clade including *S*. *fuscoirroratus* from San Cristobal and *S*. *haitensis* was dated to approximately 9.6 mya whereas the split between the San Cristobal lineage and *S*. *haitensis* was dated at 7.2 mya; the radiation of the second *S*. *fuscoirroratus* clade started about 7.9 mya. However, the confidence intervals around the estimates were large (S2 Fig.) and hence the results should be only taken as rough guidelines rather than hard evidence.

## Discussion

Most oceanic islands are colonized from the closest mainland [7, 33]. For the Galapagos Islands, this means that the common source for most colonizers is the South American mainland, which is ~1000 km away from the archipelago. Our analyses, however, clearly support an Old World origin of the Neotropic *Sphingonotus* species. The species from Galapagos and the Caribbean Islands group within the Sphingonotini with high support. In addition, the branch lengths of each island population are rather long, which supports the original designation of each island population as a distinct species or subspecies [28] despite limited phenotypic divergence [26].

### Phylogeography of the New World *Sphingonotus* species

The inferred phylogeny interpreted against the background of contemporary species distributions lets us argue that (i) grasshoppers of the tribe Sphingonotini are mainly distributed in the Old World. However, (ii) the focal species found in the Neotropics, i.e. on the Galapagos Islands and in the Caribbean, belong to the Sphingonotini rather than to the Trimerotropini, which is the predominant tribe in the New World. Hence, our analyses reject our first hypothesis that the taxonomic assignment of the Caribbean and Galapagos species to the tribe Sphingonotini is wrong. Rather our data support the hypothesis that the Caribbean (i.e. Atlantic) and the Galapagos Archipelago (i.e. Pacific) species are members of the Sphingonotini.

It has been suggested that the occurrence of *Sphingonotus* on Galapagos might be the result of a recent introduction from Europe [16]. This hypothesis can be rejected as well, since the species represent rather old lineages within the genus and are much older than most Old World species and diverged prior to any potential introduction date. While the dating is very crude the resulting age estimates are more likely an underestimate than an overestimate; the divergence between the two major clades (Trimerotropini from the New World and Sphingonotini from the Old World) was here estimated at approximately 24.4 million years ago. This dating estimate is more recent (yet both estimates have overlapping 95% HPD) than the estimate derived from a more comprehensive study which dated the split between the clades at about 35 mya [23]. The same split was dated even further back (~55 mya) by a study by Chapco & Contreras [34]. The estimate derived here is therefore a minimum estimate of the age with the lineages likely being much older. The ages of the Galapagos endemics with more than 7 mya at the basis of the lineages predate the origin of the islands.

The observed relationships may be explained by long-distance dispersal via the mainland leading to the colonization of the islands with subsequent extinction on the mainland. One might even speculate that the Sphingonotini might have colonized the American continent (e.g. [23]) and later been displaced by Trimerotropini, except for the oceanic island populations. This is supported by the high age of the islands endemics predating the ages of the islands. Alternatively, the New World *Sphingonotus* species might have reached the islands via rare long-distance, trans-Atlantic dispersal events. The first colonization step was then likely to the Caribbean, which is supported by the phylogeny. A reasonable number of studies have shown trans-Atlantic dispersal of a variety of animal and plant taxa [35–38]. For example, a study by Carranza and colleagues [39] showed a case of long-distance dispersal, where *Tarentola* Geckos invaded the Caribbean from Africa [39]; South America has been colonized by *Hemidactylus* Geckos from Africa [40], and the Americas were colonized from Africa by the grasshopper genus *Schistocerca* [36].

The Galapagos lineages of *Sphingonotus* appear to be older than many of the islands and hence a previous mainland distribution with subsequent extinction appears more likely. A continental extinction of the genus would also explain the lack of monophyly of the New World Sphingonotini. However, with our data we are not able to support with confidence either of the following hypotheses: (1) the Sphingonotini had a wider New World distribution which has been largely replaced by the Trimerotropini except for relict occurrences of *Sphingonotus* on the archipelagos or (ii) the Sphingonotini of the Galapagos archipelago and Hispaniola are the result of trans-Atlantic colonization.

### Island colonization and differentiation

In the past, *Sphingonotus fuscoirroratus* from Galapagos had been divided into two species with several subspecies [28]. Subsequently, these taxa were synonymised as only limited morphological variation between island lineages was found [26]. Our analyses suggest that each island indeed has its own distinct genetic lineage which supports the original species or subspecies status. The extent of genetic divergence of the island populations suggests that no or very little gene flow between islands exists.

Generally, inter-island radiations are typical for the Galapagos as a result of the large distance to the mainland and the relatively high distances between most islands. This can partly be confirmed here (at least for four islands). Similar radiations on the Galapagos are known for mockingbirds (*Nesomimus*) [10], tenebrionid beetles [41], iguanas (*Conolophus*) [42], and the Galapagos lava lizards [43]. The lack of monophyly of *S*. *fuscoirroratus* due to the position of the San Cristobal lineage might be caused by insufficient resolution of the data or by extinction of true sister species on the American continent. However, another explanation might be that this island was colonized independently from the others as has been shown for the Canary Islands as well [22]. However, this hypothesis would require the assumption that both lineages converged substantially in morphology when adapting to the island habitats.

### Conclusion

Our analyses support that the Galapagos endemic *S*. *fuscoirroratus* and the Caribbean endemic *S*. *haitensis* indeed belong to the tribe Sphingonotini and we therefore reject the hypothesis that these species had been wrongly assigned to the Sphingonotini. The colonization is rather ancient which allows us to reject the hypothesis that the studied species were the result of anthropogenic translocation. However, we cannot infer with certainty if the populations are relicts of a previously more widespread distribution or the result of long-distance, trans-Atlantic dispersal. In demonstrating a close phylogenetic relationship of Galapagos endemic species to Old World taxa, this study highlights the need to include geographically distantly distributed taxa in phylogeographic studies. Following the deep genetic splits detectable for our samples from Galapagos Islands, we assume that at least three to four distinct *Sphingonotus* species exist on the archipelago. It is likely that further genetic lineages are present on other islands that had not been studied here in concert with the original designation as species and subspecies [28].

## Material and Methods

### Study species

Grasshoppers of the genus *Sphingonotus* are widely distributed across major parts of the Palaearctic and Palaeotropic regions. A supposedly close relative, the genus *Trimerotropis*, can be found exclusively in the Nearctic and Neotropic region [16, 17]. The genera *Trimerotropis* and *Sphingonotus* show strong morphological similarities; however, representatives of *Trimerotropis* are mostly larger [16, 24]. Both genera had been grouped in the tribe Sphingonotini for many decades, but recently the genus *Trimerotropis* was re-assigned to the previously erected Trimerotropini [23, 44]. Both genera are species-rich with 142 species for *Sphingonotus* and 52 Species for *Trimerotropis* [17].

### Sampling

In total, 104 individuals belonging to 44 species from four continents were included in the analyses (Table 1). Specimens were collected by hand or netted and subsequently frozen or stored in ethanol. Many samples were obtained from museums or colleagues. None of the collected species are protected and no sampling was performed on protected land aside from the Galapagos. Sampling activities on Galapagos were performed by D. Otte (ANSP, Philadelphia) and S. B. Peck (Carleton University, Ottawa, Canada) under permission of the Galapagos National Park (F. Cepeda, A. Izurieta and E. Cruz, Superintendents, Department of Forestry, Ministry of Agriculture, Republic of Ecuador). The Gomphocerinae *Cibolacris parviceps* and the Oedipodinae *Chortophaga viridifasciata* served as outgroups in all analyses. Details about all individuals collected and used for this study are given in Table 1.

### Molecular analyses

Genomic DNA was extracted from dried or ethanol preserved hind leg muscle tissue using the Qiagen DNeasy Blood and Tissue Kit (Qiagen, Inc., Valencia, CA) following the manufacturer’s protocol for tissue samples. We amplified two mitochondrial and one nuclear gene fragment using a standard PCR protocol. Primers for the mitochondrial NADH Dehydrogenase subunit 5 (ND5) were obtained from Su and colleagues [45] and for COI from Husemann and colleagues [23]. The primers for Histone 3 (H3) were taken from Colgan and colleagues [46]. PCR reactions were performed using the following setup: 36.6 μl of diH_2_O, 6 μl of 10 x PCR buffer (reaction concentration 1x), 4.8 μl of dNTP mixture (0.2 μM each), 0.6 μl of DyNAzyme DNA Polymerase (1.2 U, Finnzymes, USA), 3 μl of each primer (0.5 μM, Integrated DNA technologies, USA) and 6 μl of DNA template adding up to a total volume of 60 μl. Amplification conditions were as follows: 94°C for 3 min, followed by 30 cycles of 94°C for 1 min denaturation, 48–57°C 1 min annealing and 72°C for 2 min elongation, with a final elongation step at 72°C for 10 min.

PCR products were visualized on a 1% agarose gel stained with Gel Red (0.1x, Biotium, USA and purified using Solid-phase Reversible Immobilization (SPRI) [47] with carboxylated magnetic beads (Bangs Laboratories, USA) and a 96-Ring SPRIplate (Agencourt, USA). The purified PCR products were sequenced at the Yale Sequencing Facility (New Haven, CT, USA). All sequences were deposited in Genbank; accession numbers are given in Table 1.

### Phylogenetic analyses

Sequences were inspected, trimmed and aligned using the MAFFT algorithm in Geneious 5.0.3 [48]. Further we used sequences from previous studies [18–20, 22, 23]. All genes were subsequently analyzed as combined data set. In a first step we identified the best partitioning scheme treating codon positions separately and determined the most suitable substitution models using PartitionFinder v.1.1.1 [49]. We performed two runs of PartitionFinder, one including the models implemented in MrBayes and one including the models implemented in BEAST. We then analyzed the concatenated partitioned data set with MrBayes v.3.1.2 [50]. We ran MrBayes for 50 million generations sampling every 5000 generations. A burn-in of 25% of trees was discarded before constructing a consensus tree. In addition we used BEAST v. 1.8.0 [51] to analyze the data in a supertree framework. The input file for BEAST was setup with BEAUti v. 1.8.0 (implemented in the BEAST package). We used the partitioning scheme from PartitionFinder to link the substitution models. The clock models were linked for mitochondrial genes. The trees were linked for all data. We used the Yule prior as recommended for analyses at species and genus levels and ran the analyses for 100 million iterations sampling every 10,000 iterations. The log-files were checked in Tracer v.1.5 [52] to check for convergence. A burn-in of 1000 trees was discarded before generating a consensus tree. All trees were visualized using FigTree v.1.3.1 [53].

In addition we obtained coarse estimates of divergence dates by applying a molecular clock approach. We used published substitution rates of 0.0113 for ND5 [23] and 0.01 for COI estimating the rate for H3 and applied a strict clock in BEAST v.1.8.0 [51]. No better calibration was possible as no suitable fossil data is available and using island ages as calibration points appeared inappropriate considering that we intended to estimate the divergence times of island lineages. The analysis was run for 100 million generations sampling every 10,000 generations. Trees were summarized with TreeAnnotator and visualized with FigTree.

In a last step we obtained evidence for the origin of the Galapagos taxa by using statistical DIVA and Bayes-Lagrange analyses as implemented in RASP v.3.0 [54]. We used the trees generated by our BEAST run as input and defined the geographic areas as follows: A—N America, B—Africa (including Cape Verde), C—Europe (including the Canary Islands), D—Galapagos Islands, E—Caribbean, F—Asia, G—S America. The maximum areas per node were set as 2.

## Supporting Information

S1 Fig Results from S-DIVA analysis in RASP v.3.0 (Yu et al. 2010). We used the trees generated by our BEAST run as input and defined the geographic areas as follows: A—N America, B—Africa (including Cape Verde), C—Europe (including the Canary Islands), D—Galapagos Islands, E—Caribbean, F—Asia, G—S America. The maximum areas per node were set as 2. Values represent posterior probabilities.
(DOC)

S2 Fig Divergence time estimates obtained from a molecular clock analysis in BEAST v.1.8.0 (Drummond et al. 2012). We used published substitution rates of 0.0113 for ND5 (Husemann et al. 2012) and 0.01 for COI estimating the rate for H3 and applied a strict clock. The analysis was run for 100 million generations sampling every 10,000 generations. Trees were summarized with TreeAnnotator and visualized with FigTree. Numbers are divergence times in million years. The bars represent the 95% HPDs of age estimates.
(DOC)
