# Supplementary material for: Molecular Evidence for an Old World Origin of Galapagos and Caribbean Band-Winged Grasshoppers (Acrididae: Oedipodinae: Sphingonotus)
Source: PLoS One. 2015 Feb 18;10(2):e0118208. doi: 10.1371/journal.pone.0118208 (PMC4334964; doi:10.1371/journal.pone.0118208)

S1 Figure: Results from S-DIVA analysis in RASP v.3.0 (Yu et al. 2010). We used the trees generated by our BEAST run as input and defined the geographic areas as follows: A – N America, B – Africa (including Cape Verde), C – Europe (including the Canary Islands), D – Galapagos Islands, E – Caribbean, F – Asia, G – S America. The maximum areas per node were set as 2. Values represent posterior probabilities.

Yu, Y., Harris, A.J., He, X.J., 2011. RASP (Reconstruct Ancestral States in Phylogenies). version 2.0 beta. <http://mnh.scu.edu.cn/soft/blog/RASP/>


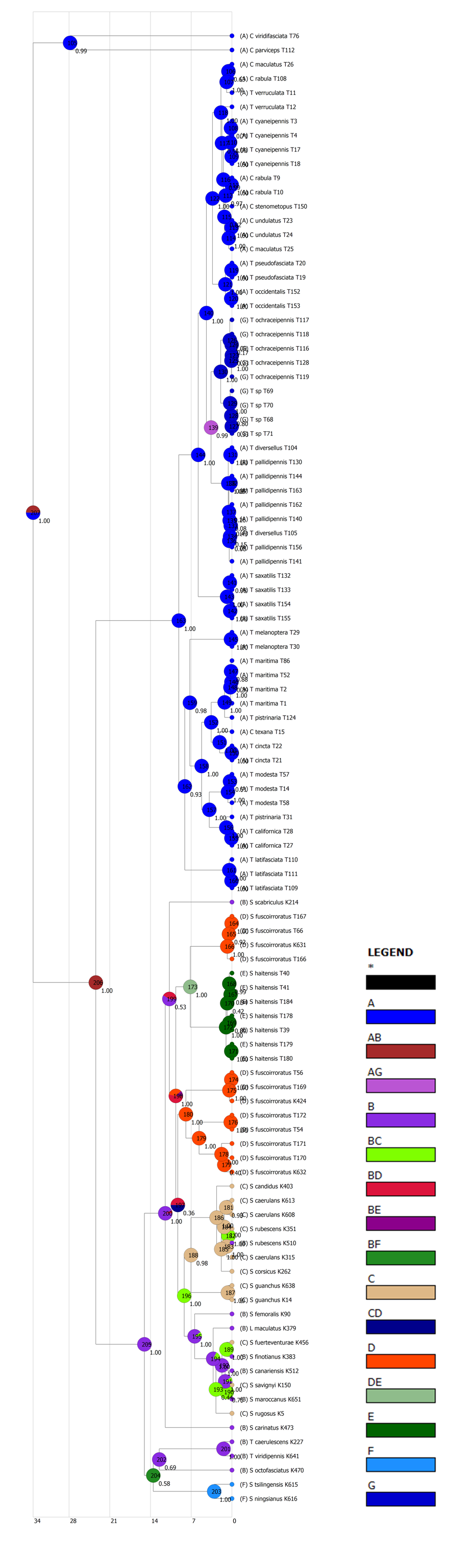

Supplement: S1 Fig — We used the trees generated by our BEAST run as input and defined the geographic areas as follows: A—N America, B—Africa (including Cape Verde), C—Europe (including the Canary Islands), D—Galapagos Islands, E—Caribbean, F—Asia, G—S America. The maximum areas per node were set as 2. Values represent posterior probabilities. (DOC) [file pone.0118208.s001.doc]
