# Supplementary material for: Molecular Evidence for an Old World Origin of Galapagos and Caribbean Band-Winged Grasshoppers (Acrididae: Oedipodinae: Sphingonotus)
Source: PLoS One. 2015 Feb 18;10(2):e0118208. doi: 10.1371/journal.pone.0118208 (PMC4334964; doi:10.1371/journal.pone.0118208)
Supplement: S2 Fig — We used published substitution rates of 0.0113 for ND5 (Husemann et al. 2012) and 0.01 for COI estimating the rate for H3 and applied a strict clock. The analysis was run for 100 million generations sampling every 10,000 generations. Trees were summarized with TreeAnnotator and visualized with FigTree. Numbers are divergence times in million years. The bars represent the 95% HPDs of age estimates. (DOC) [file pone.0118208.s002.doc]

S2 Figure. Divergence time estimates obtained from a molecular clock analysis in BEAST v.1.8.0 (Drummond et al. 2012). We used published substitution rates of 0.0113 for ND5 (Husemann et al. 2012) and 0.01 for COI estimating the rate for H3 and applied a strict clock. The analysis was run for 100 million generations sampling every 10,000 generations. Trees were summarized with TreeAnnotator and visualized with FigTree. Numbers are divergence times in million years. The bars represent the 95% HPDs of age estimates.

Drummond AJ, Rambaut A (2007) BEAST: Bayesian evolutionary analysis by sampling trees. BMC Evolutionary Biology 7: 214.

Husemann M, Namkung S, Habel JC, Danley PD, Hochkirch A (2012) Phylogenetic analyses of band-winged grasshoppers (Orthoptera, Acrididae, Oedipodinae) reveal convergence of wing morphology. Zoologica Scripta 41: 515-526.
